# Aortic growth after arch reconstruction with patch augmentation: a 2-decade experience

**DOI:** 10.1093/icvts/ivad210

**Published:** 2023-12-22

**Authors:** Dominic P Recco, Shannen B Kizilski, Reena M Ghosh, Breanna Piekarski, Ashwin Prakash, David M Hoganson

**Affiliations:** Department of Cardiac Surgery, Boston Children’s Hospital, Boston, MA, USA; Harvard Medical School, Boston, MA, USA; Department of Cardiac Surgery, Boston Children’s Hospital, Boston, MA, USA; Harvard Medical School, Boston, MA, USA; Harvard Medical School, Boston, MA, USA; Department of Cardiology, Boston Children’s Hospital, Boston, MA, USA; Department of Cardiac Surgery, Boston Children’s Hospital, Boston, MA, USA; Harvard Medical School, Boston, MA, USA; Department of Cardiology, Boston Children’s Hospital, Boston, MA, USA; Department of Cardiac Surgery, Boston Children’s Hospital, Boston, MA, USA; Harvard Medical School, Boston, MA, USA

**Keywords:** Cardiovascular surgery, Congenital heart disease, Aortic arch, Arch reconstruction, Patch augmentation

## Abstract

**OBJECTIVES:**

Optimal aortic sizing during aortic arch reconstruction remains unknown. Negative effects of arch under- or oversizing are well-published. We aimed to characterize longitudinal aortic growth after patch-augmented arch reconstruction to identify the initial reconstructed arch size that results in normal mid-term arch dimensions.

**METHODS:**

Single-centre, retrospective review of infants undergoing Damus-Kaye-Stansel (DKS) or non-DKS patch-augmented aortic arch reconstruction between 2000 and 2021. Ascending aorta, proximal and distal transverse arch, aortic isthmus (AIsth) and descending aorta dimensions were measured in postoperative echocardiograms (<3 months from index operation) and cross-sectional imaging (>12 months). Longitudinal changes to aortic dimensions and *z*-scores were analysed. Secondary outcomes included reintervention, valve and ventricular function, mortality and transplantation.

**RESULTS:**

Fifty-four patients (16 DKS, 38 non-DKS) were included. At 6.3 [2.2, 12.0]-year follow-up, all aortic segments grew significantly in both groups, while *z*-scores remained unchanged except for non-DKS proximal and distal transverse arch *z*-scores, which significantly increased (*P* < 0.05 each). When stratified by initial postoperative *z*-score (*z* < −1, −1 ≤ *z* ≤ 1, *z* > 1), non-DKS patients with initial AIsth *z*-score <−1 had a final *z*-score significantly smaller than both the targeted *z*-score zero (*P* = 0.014) and final *z*-score in a group with initial postoperative *z*-score ±1 (*P* = 0.009). Valve and ventricular function remained stable. Eighteen patients required reintervention, 1 died and 1 underwent transplant.

**CONCLUSIONS:**

Over mid-term follow-up, aortic growth after arch reconstruction with patch augmentation was proportional when repaired to normal *z*-score dimensions, aside from proximal transverse arch, which disproportionately dilated. AIsth undersizing prevailed mid-term and trended towards a higher reintervention rate. Initial reconstruction between *z*-score 0 and +1 resulted in maintenance of that *z*-score size at mid-term follow-up. Overall, it is crucial to achieve targeted aortic sizing at index operation to maintain appropriate aortic dimensions over time and reduce reintervention risk with specific focus on the AIsth.

**Figure:**
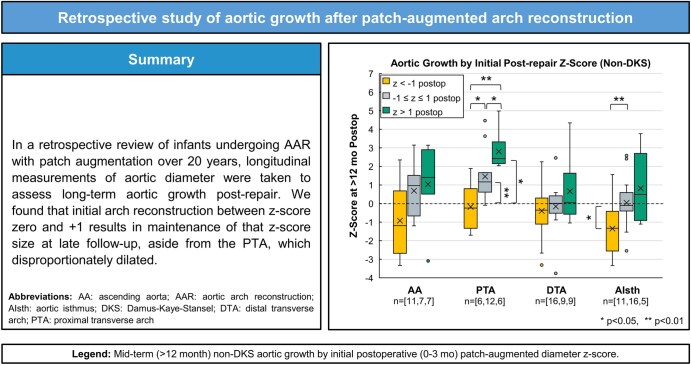

## INTRODUCTION

Reconstruction of the hypoplastic aortic arch (HAA) is a critical step in congenital cardiac procedures including stage I palliation and coarctation of the aorta repair with significant arch hypoplasia. HAA repair often involves patch augmentation to achieve normal aortic anatomy and haemodynamics. To date, the optimal aortic size and geometry at index repair remains unknown [1, 2]. Although many surgeons target *z*-score zero diameter for the ascending aorta (AA) and transverse arch, the most important outcome is not immediate postoperative size, but arch size over time. As the patch material incorporates into native tissue and somatic growth occurs, the goal is for normal arch size long-term. It is important to understand how arch dimensions post-repair dictate arch growth as the child ages. This would enable surgeons to target a particular arch size that achieves adequate short- and long-term aortic dimensions.

The consequences of late aortic mis-sizing due to inadequate initial aortic arch reconstruction (AAR) are well-studied. Long-term aortic undersizing leaves residual obstruction, which is associated with increased afterload that negatively affects ventricular function and ventriculo-arterial coupling [3, 4], and leads to relatively high reintervention rates for recoarctation [5]. Even mild persistent arch hypoplasia is associated with markedly increased risk of late systemic hypertension [6]. Conversely, aortic oversizing risks pulmonary artery compression, impedance mismatch and inefficient blood flow [7–10]. Additionally, altered aortic arch shape has important associations with poor ventricular outcomes and higher cavopulmonary pressures in AAR patients [7, 11, 12] along with decreased aortic distensibility and abnormal flow profile [13, 14]. Specifically, Quail demonstrated associations between aortic diameter and pathologic wave reflections independent of 3D curvature, suggesting that calibre is more clinically relevant than geometry [15]. Furthermore, they found that abnormal wave reflections are associated with ventricular hypertrophy after coarctation repair [10], linking repaired aortic diameter to functional outcomes.

Although there are well-established bounds that constitute an undersized or oversized arch repair, there is a paucity of literature regarding longitudinal aortic growth after AAR to determine how initial postoperative dimensions relate to aortic size at late follow-up. Eliciting this information will enable surgeons to determine a target reconstructive size that will achieve acceptable short- and long-term arch dimensions and provide improved long-term outcomes. This study was conducted with the aim of determining longitudinal aortic growth characteristics after AAR with patch augmentation and evaluating the related clinical outcomes.

## MATERIALS AND METHODS

### Ethical statement

The study was approved by Boston Children’s Hospital Institutional Review Board (IRB-P00041620) with the need for consent waived.

### Study design and patients

A single-centre, retrospective review was conducted of all paediatric patients undergoing AAR with patch augmentation between January 2000 and December 2021. Patients who underwent AAR without patch augmentation (i.e. end-to-end anastomosis, transplantation or synthetic tube-graft repair) or salvage procedures were excluded. Due to fundamental differences in arch geometry, surgical goals and physiology, patients were divided into Damus-Kaye-Stansel (DKS) and non-DKS groups. The DKS group included all patients who previously underwent DKS reconstruction prior to or at the index procedure. All outcome measures are reported separately for these groups without the goal of comparing between the groups but separately reporting outcomes for the 2 groups.

**Figure 1:**
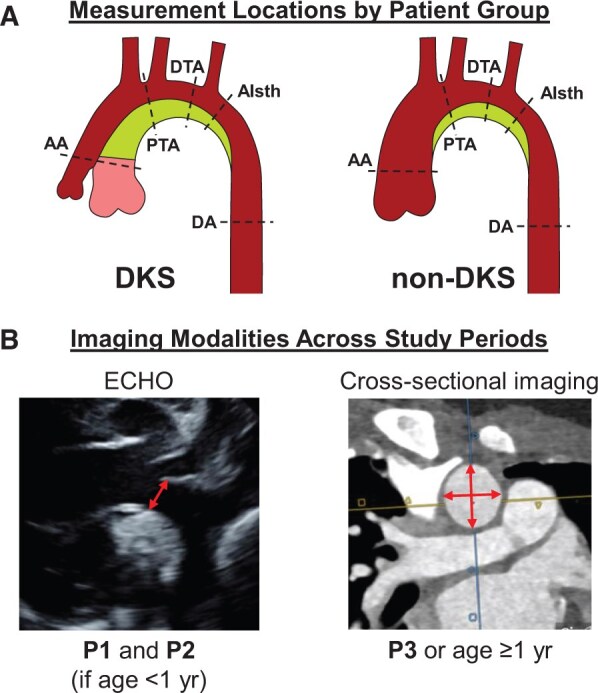
(**a**) Aortic dimensions were measured at 5 predefined locations: (1) ascending aorta (AA), (2) proximal transverse arch (PTA), (3) distal transverse arch (DTA), (4) aortic isthmus (AIsth) and (5) descending aorta (DA) for both Damus-Kaye-Stansel (DKS) and non-DKS patient groups. (**b**) Aortic dimensions were assessed using certain imaging modalities at 3 postoperative periods: <3 months (P1), between 3 and 12 months (P2) and >12 months (P3) from index procedure. Echocardiography was used during P1 and P2 in patients <1 year old. Cross-sectional imaging was exclusively used during P3 and in infants >1 year old and was the preferred modality across all periods.

Outcome measures were assessed at 3 postoperative periods: <3 months (P1), between 3 and 12 months (P2) and >12 months (P3) from index procedure (Fig. 1). If a patient had multiple imaging studies within a given period, the latest study was used. All included patients were required to have P1 and P3 imaging, but not all had P2 imaging. Specific exclusion criteria for aortic imaging are described in the ‘Aortic measurements’ section. Institutional databases were queried, and baseline population data, surgical details, postoperative complications and clinical events were collected. Of note, follow-up time was based on the time at which the patient last underwent cross-sectional imaging.

### Outcome measures

The primary end-point was aortic growth over time, described by the change in diameter and corresponding *z*-score at 5 predefined locations along the aorta over the follow-up period. The *z*-score was calculated from the patient’s body surface area (BSA), using our institution’s previously derived regression model [16] for the AA, distal transverse arch (DTA) and aortic isthmus (AIsth) locations. The Paediatric Heart Network *z*-scores were used for the proximal transverse arch (PTA) [17], and neither database had descending aorta (DA) *z*-scores. Definitions for each of these aortic measurements are described in the next section. Secondary outcomes included intensive care unit (ICU) and hospital length of stay (LOS), reinterventions (see ‘Reintervention’ section), left ventricular outflow tract obstruction (LVOTO) intervention, all-cause mortality (30 days and 1 year) and transplantation. Echocardiography (ECHO)-derived secondary outcomes included systemic ventricular function, end-diastolic volume (EDV) and mass-to-volume ratio and systemic atrioventricular valve (AVV) function (see ‘Echocardiography measurements’ section).

### Aortic measurements

To obtain highly reliable aortic measurements, acceptable imaging modalities were established based on patient age. Although transthoracic echocardiography (TTE) can provide excellent aortic arch assessment, particularly in infants, it is dependent on adequate acoustic windows. Cardiac magnetic resonance (CMR) imaging is ideally suited for serial imaging of the aortic arch, as it enables 3D visualization of the region of interest and facilitates highly accurate and reproducible vascular measurements [18]. Cardiac computed tomography (CCT) offers similar benefits to CMR with increased spatial resolution but in the setting of a radiation dose. With TTE deemed relatively inaccurate compared to CMR/CCT in children over 1 year old [18, 19], it was used only for P1 and P2 aortic measurements in patients <1 year of age. For P3 measurements, and for any period when a patient was >1 year of age, cross-sectional imaging was utilized. Any patients without both P1 and P3 imaging that met these requirements were excluded. Lastly, patients with cross-sectional imaging of poor quality and/or resolution, impeding accurate aortic measurements, were excluded.

ECHO measurements were performed by a paediatric cardiologist (Reena M. Ghosh) using Merge Cardio. ECHO was used to evaluate the aortic dimensions at the P1 and P2 timeframes. Cross-sectional imaging measurements were performed by a surgical resident (Dominic P. Recco) trained by a paediatric cardiologist who specializes in cardiac cross-sectional imaging (Ashwin Prakash). Dimensions were measured orthogonal to the vessel’s longitudinal axis utilizing 3D multiplanar reformation in Circle CVI^42^. Cross-sectional imaging was the preferred modality to evaluate the aortic dimensions at all postoperative periods (P1, P2 and P3). CCT/MRI was available and used for 2 patients during the P1 period [2 non-DKS patients (5%)], 11 patients during P2 [3 DKS (19%) and 8 non-DKS (21%)] and all 58 patients during P3. Aortic growth was assessed at discrete locations across the aorta by the relative increase or reduction in dimension and *z*-score over time. Aortic dimensions were measured at 5 predefined locations:

1. AA: diameter at the level of the right pulmonary artery.
2. PTA: diameter between the brachiocephalic trunk take-off and left common carotid artery. If variation in head vessel branching was present, PTA was defined as the diameter between the take-off of the right and left common carotid arteries.
3. DTA: narrowest diameter between the take-off of the left common carotid and left subclavian arteries.
4. AIsth: diameter just distal to the left subclavian artery origin.
5. DA: diameter at the level of the left atrium.

Patients were further stratified by smaller (*z*-score < −1), middle (−1 ≤ *z*-score ≤ 1) and larger (*z*-score > 1) segmental aortic *z*-scores at P1 to assess differential growth across these groups. This analysis was performed to identify how initial postoperative size impacts long-term aortic size and growth, further eliciting a target reconstructive size range that achieves acceptable short- and long-term arch dimensions. Secondary outcomes were also compared across groups to determine a target size that generates optimal functional results for use in patch planning.

### Reintervention

Reintervention was defined as any endovascular or surgical procedure performed on the aortic arch during the study period following the index operation. Examples include angioplasty, stenting, redo patch augmentation and/or arch replacement. For patients who underwent multiple reinterventions in the postoperative period, only the initial procedure was considered for analysis. Time to first reintervention was recorded.

### Echocardiography measurements

ECHO-derived secondary outcome measurements were recorded during the same time periods (P1, P2, P3) as the aortic diameters. For P1 and P2, the TTE used for aortic measurements was employed. For P3, the ECHO closest to the date of the cross-sectional imaging was used. As a subset of ECHOs were missing quantitative data, a coding system was devised to translate the available qualitative descriptions of the 4 ECHO measurements of interest into comparable quantities. The coding system was as follows: 0 = normal, 1 = trivial, 2 = mild, 3 = mild to moderate, 4 = moderate, 5 = moderate to severe and 6 = severe. The reported values written as median [Q1, Q3] were calculated utilizing this scheme.

### Statistical analysis

Baseline characteristics, operative details, and primary and secondary outcomes were summarized using median and interquartile range [Q1, Q3] or frequency counts and percentages. DKS and non-DKS groups were analysed separately, as comparison between these groups would not provide any meaningful clinical insights. Diameter versus BSA data were fit to an exponential model ln(*y*) = (*a* + *b* × ln(*x*)), where ‘*y*’ is the aortic segment diameter, ‘*a*’ is the intercept (cited constant), ‘*b*’ is β (cited constant) and ‘*x*’ is the BSA [20], for qualitative comparison to the population *z*-score zero curve. Given the small sample size and nonparametric nature of the data, within-group analysis was performed with the Wilcoxon signed-rank test, and a one-sample Wilcoxon test was used to assess significant difference between median *z*-scores and 0. Between-group analysis was performed with the Kruskal–Wallis test followed by post hoc Wilcoxon rank-sum tests as needed with Bonferroni correction for multiple comparisons. Comparisons between groups for select secondary outcomes were performed using Wilcoxon rank-sum test for continuous variables and Fisher’s exact test for categorical variables. Kaplan–Meier analysis was conducted to evaluate time to reintervention, survival and transplant-free survival. All analyses were conducted using MATLAB 2021b, and *P*-values <0.05 were considered statistically significant.

## RESULTS

### Patient characteristics and operative details

**Figure 2:**
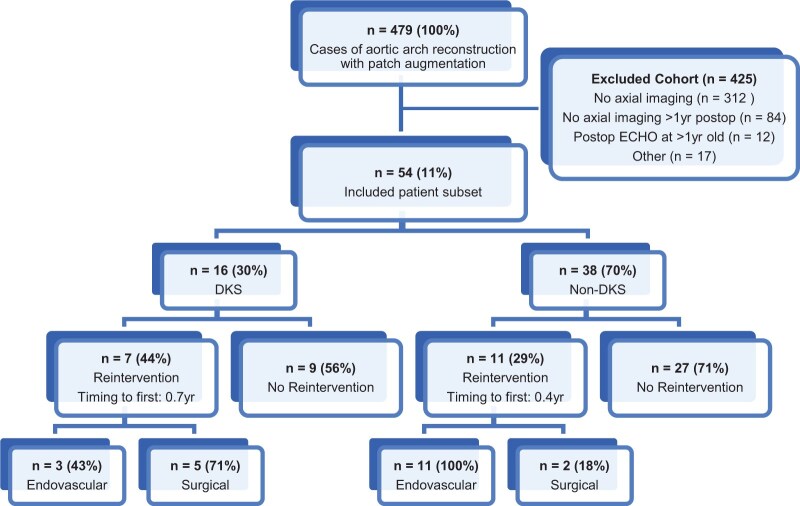
CONSORT flow diagram with detail of aortic arch reintervention rate and reintervention type (endovascular versus surgical) by patient subgroup (DKS and non-DKS). DKS: Damus-Kaye-Stansel.

Of 479 eligible patients who underwent AAR with patch augmentation, a total of 54 patients met inclusion criteria (Fig. 2), divided into DKS (*n* = 16) and non-DKS (*n* = 38) groups. In the DKS group, weight and age at surgery were 5.4 [4.4, 6.9] kg and 166 [117, 194] days, with 3 patients (19%) operated on as neonates. Most patients in this group (69%) held a combination of cardiac diagnoses, with 4 (25%) having isolated hypoplastic left heart syndrome/single ventricle variant and 1 (6%) isolated HAA/arch obstruction. Four patients (25%) underwent previous surgical arch intervention, while 7 (44%) previously underwent both surgical and endovascular arch intervention. Eleven patients (69%) underwent patch aortoplasty alone at the time of index AAR, and 14 patients (88%) required concomitant procedures. Of note, it is standard of practice at our institution to perform coarctectomy in all DKS and non-DKS patients with aortic coarctation, which entailed resection of the aorta at the level of coarctation with resection of all ductal tissue and posterior shelf, with or without interdigitation, followed by the preferred anastomotic technique (e.g. end-to-end, extended end-to-end, arch advancement). Pulmonary homograft was the most frequently used patch material (63%), followed by bovine pericardium (25%). Follow-up time to latest cross-sectional imaging was 3.3 [2.1, 10.1] years.

In the non-DKS group, weight and age at surgery were 3.3 [2.9, 3.6] kg and 6.5 [4, 10] days, with 32 patients (84%) operated on as neonates. Most patients (76%) held a combination of cardiac diagnoses, with 3 (8%) having coarctation of the aorta and HAA, 4 (11%) isolated HAA/arch obstruction and 2 (5%) isolated IAA. Four patients (11%) had a secondary cardiovascular diagnosis of William’s syndrome. None of the non-DKS patients had history of previous surgical or endovascular arch intervention. Twenty-seven patients (71%) underwent end-to-end anastomosis or interdigitating end-to-end anastomosis at the time of index AAR, and 36 patients (95%) required concomitant procedures. Pulmonary homograft patch was used in 25 patients (66%) and autologous pericardium used in 12 (32%). Follow-up time was 8.6 [2.9, 13.7] years. Additional patient characteristics and operative details are summarized in Table 1.

**Table 1:** Baseline characteristics and operative details

	Total, 54 (100%)		DKS, 16 (30%)		Non-DKS, 38 (70%)	
Baseline characteristics						
Gender, *n* (%)						
Male	26 (48)		8 (50)		18 (47)	
Female	28 (52)		8 (50)		20 (53)	
Premature, *n* (%)						
Yes	4 (7)		0 (0)		4 (11)	
No	50 (93)		16 (100)		34 (89)	
Cardiac diagnosis	Isolated	Multiple	Isolated	Multiple	Isolated	Multiple
HLHS and SV variations, *n* (%)	4 (7)	6	4 (25)	6	0 (0)	0
TGA, *n* (%)	0 (0)	8	0 (0)	2	0 (0)	6
CoA and HAA, *n* (%)	3 (6)	23	0 (0)	4	3 (8)	19
HAA/arch obstruction, *n* (%)	5 (9)	34	1 (6)	9	4 (11)	25
IAA, *n* (%)	2 (4)	2	0 (0)	0	2 (5)	2
Other, *n* (%)	0 (0)	16	0 (0)	4	0 (0)	12
Syndromic disease, *n* (%)						
Willam’s syndrome	4 (7)		0 (0)		4 (11)	
Neonate at surgery, *n* (%)						
Yes	35 (65)		3 (19)		32 (84)	
No	19 (35)		13 (81)		6 (16)	
Age at surgery (days), median (IQR)	8.5 (5, 129)		166 (117, 194)		6.5 (4, 10)	
Weight at surgery (kg), median (IQR)	3.4 (3.0, 5.2)		5.4 (4.4, 6.9)		3.3 (2.9, 3.6)	
BSA at surgery (m^2^)	0.23 (0.21, 0.30)		0.31 (0.27, 0.36)		0.22 (0.20, 0.23)	
Cardiac surgical history, *n* (%)						
Yes	18 (33)		13 (81)		5 (13)	
No	36 (66)		3 (19)		33 (87)	
Previous arch intervention, *n* (%)						
Endovascular	0		0 (0)		0 (0)	
Surgical	4 (7)		4 (25)		0 (0)	
Both	7 (13)		7 (44)		0 (0)	
Operative details						
Procedure status, *n* (%)						
Elective	39 (72)		12 (75)		27 (71)	
Urgent	14 (26)		4 (25)		10 (26)	
Emergent	1 (2)		0 (0)		1 (3)	
Surgical technique, *n* (%)						
Patch aortoplasty only	17 (31)		11 (69)		6 (16)	
EEA	15 (28)		1 (6)		14 (37)	
Interdigitating EEA	15 (28)		2 (13)		13 (34)	
EEEA	5 (9)		2 (13)		3 (8)	
Aortic arch advancement	2 (4)		0 (0)		2 (5)	
Concomitant procedures, *n* (%)						
Yes	50 (93)		14 (88)		36 (95)	
No	4 (7)		2 (12)		2 (5)	
Patch material, *n* (%)						
Pulmonary homograft	35 (65)		10 (63)		25 (66)	
Autologous pericardium	12 (22)		0 (0)		12 (32)	
Bovine pericardium	4 (7)		4 (25)		0 (0)	
Other	3 (6)		2 (12)		1 (3)	
CPB time (min), median (IQR)	159 (135, 196)		160 (136, 187)		159 (136, 198)	
Cross-clamp time (min), median (IQR)	90 (60, 117)		54 (32, 71)		106 (82, 123)	
Circulatory arrest time (min), median (IQR)	22 (7, 39)		27 (11, 34)		19 (6, 43)	
	*N* = 42		*N* = 14		*N* = 28	

BSA: body surface area; CoA: coarctation of the aorta; CPB: cardiopulmonary bypass; DKS: Damus-Kaye-Stansel; EEA: end-to-end anastomosis; EEEA: extended end-to-end anastomosis; HAA: hypoplastic aortic arch; HLHS: hypoplastic left heart syndrome; IAA: interrupted aortic arch; IQR: interquartile range; SV: single ventricle; TGA: transposition of the great arteries.

### Longitudinal aortic diameter measurements

**Figure 3:**
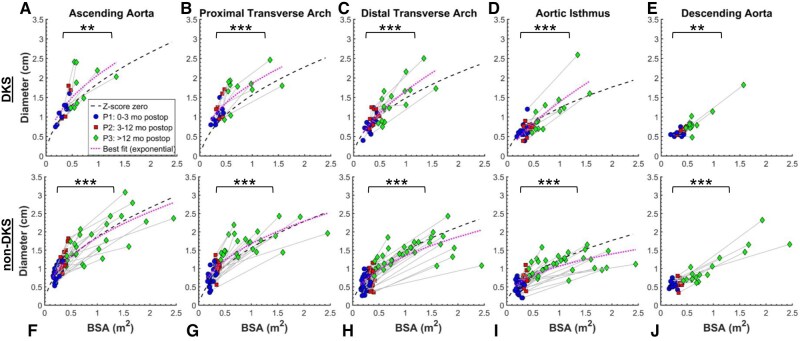
Longitudinal aortic diameter measurements by body surface area (BSA) for (**a**–**e**) Damus-Kaye-Stansel (DKS) and (**f**–**j**) non-DKS patient groups. In each panel, blue circles indicate P1 measurements (0–3 months postoperative), red squares show P2 measurements (3–12 months postoperative) and green diamonds show P3 measurements (>12 months postoperative). Black dashed lines show normative *z*-score zero values for each aortic segment, where available. Magenta dotted lines show best-fit exponential curves for the data, with *r*^2^ values shown. Brackets and stars indicate statistically significant increase in diameter between P1 and P3. (*) denotes *P* < 0.05, (**) denotes *P* < 0.01 and (***) denotes *P* < 0.001.

All aortic diameter measurements are listed in Supplementary Material, Table S1. In both groups, measurements were available at both P1 and P3 for a subset of the patient cohort at each segment location (Fig. 3a–e for DKS, Fig. 3f–j for non-DKS). In the DKS group, BSA increased from 0.32 [0.26, 0.38] m^2^ at P1 to 0.64 [0.56, 1.0] m^2^ at P3. All aortic segments grew significantly from P1 to P3 (*P* < 0.01 each). In the non-DKS group, BSA increased from 0.22 [0.20, 0.28] m^2^ at P1 to 0.96 [0.60, 1.49] m^2^ at P3. All aortic segments in this cohort also grew significantly from P1 to P3 (*P* < 0.001 each).

### Longitudinal aortic z-scores

**Figure 4:**
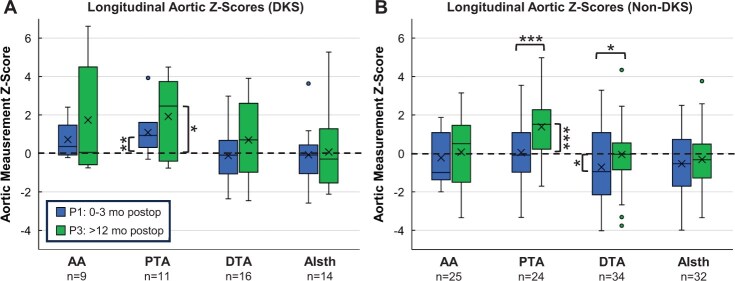
Longitudinal aortic *z*-score measurements for the ascending aorta (AA), proximal (PTA) and distal transverse arch (DTA) and aortic isthmus (AIsth) for (**a**) Damus-Kaye-Stansel (DKS) and (**b**) non-DKS patient groups. For each aortic segment, 2 boxes show *z*-score measurements for periods 1 and 3 (P1, P3). The line in each box denotes the median and the x denotes the mean. Boxes are bounded by Q1 and Q3, whiskers show the range excluding outliers and outliers are shown as dots. Brackets and stars above the boxes indicate statistically significant difference in *z*-score between P1 and P3. Brackets and stars next to a single box indicate that the median of that group was significantly different from *z*-score zero. (*) denotes *P* < 0.05, (**) denotes *P* < 0.01 and (***) denotes *P* < 0.001.

AA, PTA, DTA and AIsth diameters were converted to BSA-indexed *z*-score values; these data were not available for DA, which was excluded from further analysis. All aortic *z*-scores are listed in Supplementary Material, Table S1. In the DKS group (Fig. 4a), PTA *z*-scores at P1 and P3 were both statistically larger than *z*-score zero (for normal arch anatomy) (P1: 0.94 [0.35, 1.56], *P* = 0.003; P3: 2.47 [−0.12, 3.60], *P* = 0.024) without significant change in *z*-score between the 2 timepoints (*P* = 0.206).

In the non-DKS group, the PTA *z*-score increased significantly from −0.09 [−0.86, 1.03] to 1.52 [0.29, 2.29] (*P* < 0.001) between P1 and P3, resulting in a P3 *z*-score significantly larger than zero (*P* < 0.001). The DTA was significantly smaller than *z*-score zero at P1 at −0.95 [−2.06, 1.07] (*P* = 0.046). From P1 to P3, the DTA *z*-score increased significantly to −0.04 [−0.84, 0.53] (*P* = 0.048) (Fig. 4b).

### Stratifying aortic growth by initial postoperative z-score

**Figure 5:**
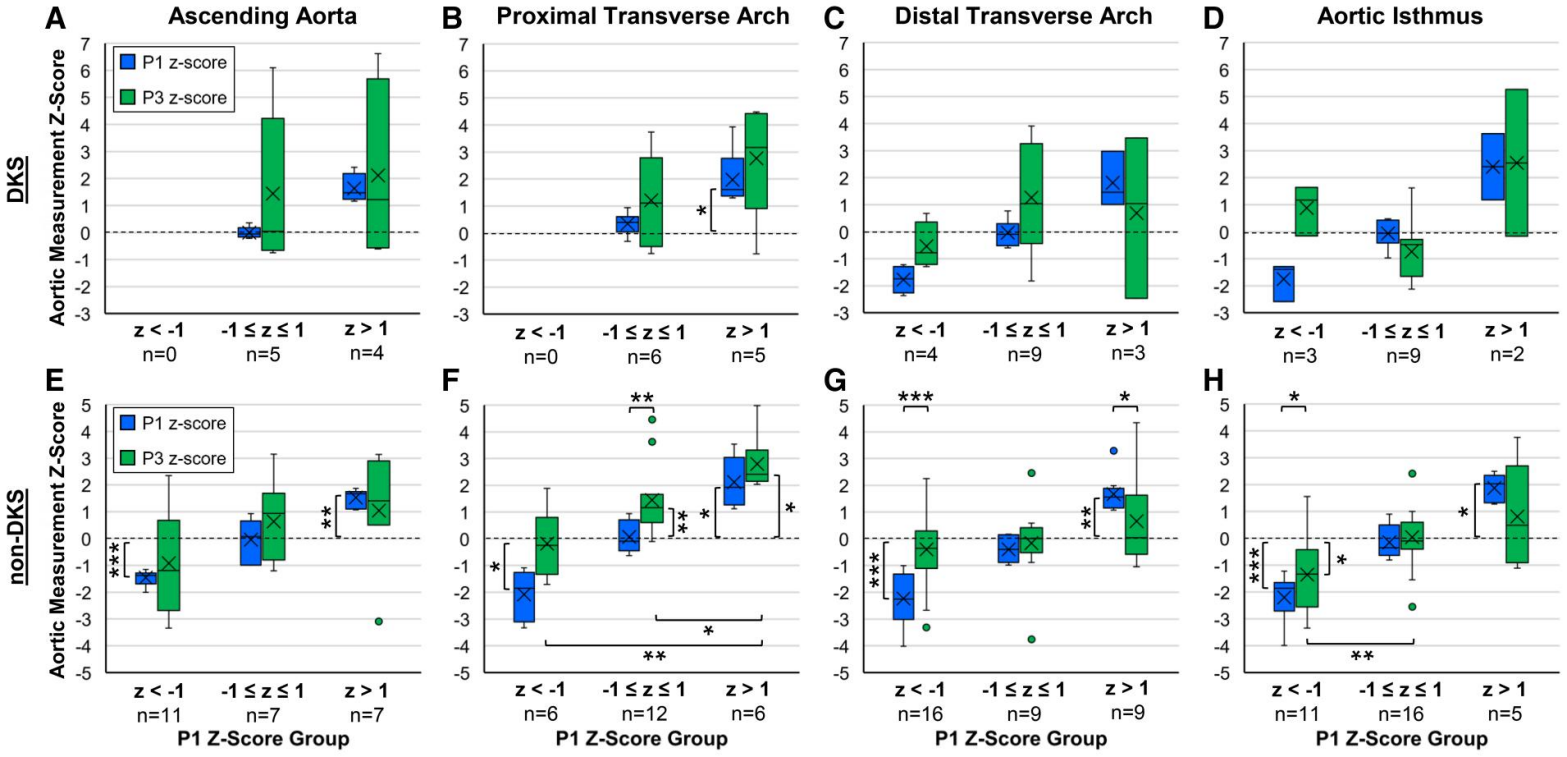
Segmental aortic *z*-score values at P1 (0–3 months postoperative) and P3 (>12 months postoperative) stratified by *z*-score at initial post-repair measurement (P1 *z*-score groups: *z* < −1, −1 ≤ *z* ≤ 1 and *z* > 1) for the ascending aorta, proximal and distal transverse arch and aortic isthmus, for (a−d) Damus-Kaye-Stansel (DKS) and (e−h) non-DKS patient groups. Numbers under each P1 *z*-score group indicate the number of patients in the respective groups. The line in each box denotes the median and the *x* denotes the mean. Boxes are bounded by Q1 and Q3, whiskers show the range excluding outliers and outliers are shown as dots. Brackets and stars above the boxes indicate statistically significant differences between P1 and P3 *z*-scores within a respective given P1 *z*-score grouping, while brackets and stars below the boxes indicate statistically significant differences between the P3 *z*-scores across the initial repair size groups. Brackets and stars next to a single box indicate that the median of that group was significantly different from *z*-score zero. (*) denotes *P* < 0.05, (**) denotes *P* < 0.01 and (***) denotes *P* < 0.001.

Patients were stratified by smaller (*z*-score < −1), middle (−1 ≤ *z*-score ≤ 1) and larger (*z*-score >1) segmental aortic P1 *z*-scores to assess differential growth across these groups (Fig. 5 and Supplementary Material, Table S2). Of note, in the DKS group, no patients fell into the smaller P1 AA or PTA groups (likely related to typical DKS geometry). When stratified by initial post-repair *z*-score, none of the P3 *z*-scores for any segment were statistically different from *z*-score zero (Supplementary Material, Table S2).

In the non-DKS group, patients were more evenly spread across the 3 P1 *z*-score groups; smaller initial post-repair *z*-scores were significantly below *z*-score zero for all locations, and larger initial post-repair *z*-scores were significantly greater than *z*-score zero (*P* < 0.05 each). Regardless of P1 *z*-score, the PTA *z*-score increased from P1 to P3, with the middle group reaching statistical significance (*P* = 0.002). The smaller group PTA P3 *z*-score (−0.25 [−1.21, 0.43]) was slightly smaller than the P3 *z*-score for the middle (1.16 [0.70, 1.66], *P* = 0.042) group and significantly smaller than the larger (2.42 [2.19, 2.76], *P* = 0.002) group. Additionally, the middle P3 *z*-score was lower than the larger repair group P3 *z*-score (*P* = 0.014), but both groups had P3 *z*-scores greater than *z*-score zero (*P* < 0.05 each).

For the DTA in the non-DKS group, the P1 smaller (*z*-score −2.25 [−2.96, −1.36]) and larger (*z*-score 1.56 [1.17, 1.84]) groups both grew disproportionately to achieve a *z*-score not different from zero (*P* < 0.05 for both) by P3. At P3, DTA *z*-scores were −0.36 [−1.10, 0.28] for the smaller group, 0.01 [−0.34, 0.33] for the middle group and 0.04 [−0.44, 1.57] for the larger group (*P* = 0.309 for inter-group comparison). Similarly, smaller and larger AIsth groups trended closer to *z*-score zero by P3, however, unlike all other stratified P3 results, the smaller AIsth repair group maintained a *z*-score at P3 that was significantly less than zero, with a value of −1.33 [−2.52, −0.47] (*P* = 0.014).

### Secondary outcomes

In the DKS group, there was 100% 30-day survival. Survival at 15 years was 93% [95% confidence interval (CI): 61, 99] (Supplementary Material, Fig. S1) with 1 patient (6%) requiring transplantation. Postoperative ICU and hospital LOS were 5.7 [3.1, 8.3] and 12.7 [8.1, 24.6] days. Freedom from arch reintervention (surgical or endovascular) at 15 years was 40% (95% CI: 8, 71) (Supplementary Material, Fig. S2) with median timing to first reintervention of 0.7 [0.6, 8.2] years after index surgery (Fig. 2). Three patients (19%) developed LVOTO requiring intervention. Following the predefined scoring system, the AVV function score, systemic ventricular function and EDV scores remained constant from P1 to P3. Systemic ventricle mass-to-volume ratio score decreased from 3 [2, 4.5] at P1 to 2 [2, 2] at P3.

In the non-DKS group, there was 100% 30-day survival. Survival at 15 years was 97% (95% CI: 83, 100) (Supplementary Material, Fig. S1), and no patients underwent transplantation. Postoperative ICU and hospital LOS were 7.4 [6.0, 10.8] and 17.4 [10.2, 23.0] days. Freedom from arch reintervention at 15 years was 66% (95% CI: 44,80) (Supplementary Material, Fig. S3) with median timing to first reintervention of 0.4 [0.3, 0.8] years (Fig. 2). When stratified by P1 AIsth *z*-score, 5/11 (45%) patients in the smaller repair group required reintervention, 4/16 (25%) in the middle group and 0/5 (0%) in the larger group. Stratifying P1 AIsth *z*-score into <0 vs ≥0 resulted in a statistically insignificant higher reintervention rate in the *z*-score <0 group compared to the *z*-score ≥0 group (8/22 [36%] vs 1/10 [10%]; *P* = 0.132). Eleven patients (29%) required LVOTO intervention. AVV function, systemic ventricular function and systemic ventricular EDV score remained relatively consistent P1 to P3. Systemic ventricle mass-to-volume ratio score decreased from 1 [0, 3.75] at P1 to 0 [0, 0.25] at P3. Additional postoperative outcomes are summarized in Table 2. No significant correlations were found between aortic *z*-scores and any of the secondary outcomes in either group due to the low statistical power of this study.

**Table 2:** Postoperative outcomes

Postoperative outcomes	Total, 54 (100%)	DKS, 16 (30%)	Non-DKS, 38 (70%)
Follow-up (years), median (IQR)	6.3 (2.2, 12.0)	3.3 (2.1, 10.1)	8.6 (2.9, 13.7)
Follow-up index, median (IQR)	0.76 (0.53, 0.90)	0.63 (0.41, 0.78)	0.81 (0.54, 0.91)
Postoperative ICU LOS (days), median (IQR)	7.0 (4.9, 10.4)	5.7 (3.1, 8.3)	7.4 (6.0, 10.8)
Postoperative hospital LOS (days), median (IQR)	16.8 (8.8, 23.3)	12.7 (8.1, 24.6)	17.4 (10.2, 23.0)
15-Year freedom from arch reintervention (%)	57 (95% CI: 38, 72)	40 (95% CI: 8, 71)	66 (95% CI: 44, 80)
Endovascular	70 (95% CI: 54, 82)	81 (95% CI: 51, 93)	66 (95% CI: 44, 80)
Surgical	81 (95% CI: 61, 91)	47 (95% CI: 9, 79)	95 (95% CI: 80, 99)
Timing to first reintervention (years), median (IQR)	0.6 (0.3, 3.9)	0.7 (0.6, 8.2)	0.4 (0.3, 0.8)
Development of LVOTO requiring intervention, median (IQR)			
Yes	14 (26)	3 (19)	11 (29)
No	40 (74)	13 (81)	27 (71)
Systemic AVV function, median (IQR)			
0–3 months	1 (0, 2)	2 (1, 2)	1 (0, 2)
>12 months	1 (1, 2)	2 (1, 3)	1 (0, 2)
Systemic ventricular function, median (IQR)			
0–3 months	0 (0, 1)	0.5 (0, 2)	0 (0, 0)
>12 months	0 (0, 1)	0.5 (0, 2)	0 (0, 0)
Systemic ventricle EDV, median (IQR)			
0–3 months	0 (0, 2)	2 (2, 2)	0 (0, 0)
>12 months	2 (0, 2)	2 (2, 2)	0 (0, 2)
Systemic ventricle mass-to-volume ratio, median (IQR)			
0–3 months	2 (0, 4)	3 (2, 4.5)	1 (0, 3.75)
>12 months	0 (0, 2)	2 (2, 2)	0 (0, 0.25)
Survival (%)			
30 days	100	100	100
15 yeard	96 (95% CI: 85, 99)	93 (95% CI: 61, 99)	97 (95% CI: 83, 99)
Transplant free	98 (95% CI: 88, 100)	93 (95% CI: 61, 99)	100

AVV: atrioventricular valve; CI: confidence interval; DKS: Damus-Kaye-Stansel; EDV: end-diastolic volume; ICU: intensive care unit; IQR: interquartile range; LOS: length of stay; LVOTO: left ventricular outflow tract obstruction.

## DISCUSSION

This study resulted in important numerical understanding of the growth and geometric changes of patch-augmented aortas over time. The non-DKS and DKS cohorts were evaluated in parallel without comparison but deriving separate analyses of how to approach surgical repair of each cohort independently. In the non-DKS cohort, the *z*-score size for all 4 areas of the arch at initial repair had a median *z*-score of less than zero. The degree of proximal extension of the patch likely varied, so the AA size may reflect the native AA size in many of the patients. However, an important finding is that the AIsth *z*-score median was nearly −1 and that segment *z*-score did not significantly change as the children grew. When stratified by P1 *z*-score, if the AIsth *z*-score at initial reconstruction was below −1, it was still below −1 at late follow-up. Additionally, patients with AIsth size of *z*-score <0 at initial reconstruction had a reintervention rate of 36% compared to 10% in patients with initial AIsth size of *z*-score ≥0. Although this difference was not statistically significant due to small sample size, it likely represents an opportunity to aim for a larger arch size in the reconstructed isthmus area to avoid a chronically small AIsth with risk of reintervention. Additionally, the authors support performing a formal coarctectomy with any preferred surgical approach if an aortic coarctation is present to minimize the risk of postoperative re-obstruction and reintervention. Although the DTA was also small at initial reconstruction with median *z*-score −1, it increased in average size to *z*-score zero at late follow-up. Stratification by initial post-repair *z*-scores showed that patients with smaller and larger DTA exhibited disproportional growth (*z*-score increase and decrease, respectively) to reach *z*-score zero by P3. In contrast, PTA increased in *z*-score regardless of initial size. If the PTA was *z*-score zero (±1) at initial reconstruction, it was *z*-score +1 at late follow-up. However, the PTA greater than *z*-score +1 at initial reconstruction averaged nearly *z*-score +2.5 at late follow-up. It is likely a combination of factors including patch material, native aortic properties and haemodynamics (i.e. turbulence, wall stress, flow velocity changes with arch curvature, etc.) that may contribute to this, but oversizing the PTA at initial reconstruction may carry a risk of disproportionate oversizing as the child grows. Any area of the arch that is oversized relative to the downstream segment elicits backward compression waves in systole, which increase left ventricle impedance and afterload [10, 15, 21]. Also, if the patients have a relatively small AA that is only augmented in the distal aspect, the PTA enlargement could be a post-stenotic dilation effect, a consequence of turbulent flow and abnormal wall stress [22, 23]. Of note, 4 patients held a diagnosis of William’s syndrome which may have stunted aortic growth, especially of the AA. Given the small percentage of patients with syndromic disease, the effect of such processes is thought to be minimal on the reported results and underlying conclusions.

In the DKS cohort, nearly all patients had AA and PTA *z*-score ≥0 at P1 due to the nature of the proximal anastomosis and typical DKS geometry. The ideal shape and size of the AA and PTA for neonatal or infant DKS reconstructions has not been elucidated, so the true target *z*-score zero in these patients is not yet known. Similar to the non-DKS group, PTA and DTA *z*-scores appeared to increase slightly from P1 to P3, though this was not statistically significant. Overall, when the AA, PTA and DTA in DKS patients were reconstructed to *z*-score zero (±1), the late follow-up averaged *z*-score +1 for each of these locations, which is quite important in light of the known consequences of late aortic mis-sizing. Targeting an initial *z*-score of 0 to 1 in these locations should then correlate with acceptable mid-term arch calibre. Initial AIsth *z*-score between ±1 resulted in a final *z*-score significantly smaller than the group initially repaired to a *z*-score of <−1. As this finding is counterintuitive, we believe this to be a consequence of the small data set and possibly the higher rate of reintervention in the smaller group and not truly a reflection of DKS growth patterns.

AAR with patch augmentation is an essential component in repair of multiple congenital cardiac anomalies. Historically, reconstruction of the hypoplastic aorta has been performed with the goal to restore relatively normal anatomy but without a full understanding of how the arch grows from the initial postoperative reconstruction size. Less than favourable long-term results have led to a multitude of different surgical approaches utilizing alternative patch materials and anastomotic techniques [24–26]. Despite concerted efforts, there is a considerable 9–37% risk of arch renarrowing requiring reintervention [5, 24–29]. Inconsistent outcomes may be linked to limited information on how the repaired arch grows over time. Mahle found that, after Norwood operation, transverse aortic diameter increased significantly over 6.5-year follow-up at a growth rate that paralleled the normal population [30]. More recently, Haller showed promising short-term outcomes with the interdigitating technique in HLHS patients who underwent staged palliation. In this cohort, there were relatively low recoarctation and reintervention rates at 2-year follow-up, and aortic growth occurred proportionally [2]. However, with only medium-term follow-up, long-term sequelae of AAR remain uncertain and the optimal target reconstructive size across aortic segments remains unknown. Although our study similarly demonstrated a trend of proportional growth longitudinally in both DKS and non-DKS cohorts, we also showed that the growth trajectory in individual patients was dependent upon the initial repair size, with different responses across each aortic region.

In this context, a prospective, patient-specific, patch-planning workflow is currently under development at our institution utilizing 3D modelling and virtual surgery to accurately predict patch dimensions required to achieve target aortic size and geometry during AAR with patch augmentation. Within this workflow, reconstructive patches are designed based on 3D models of the pathologic preoperative anatomy and targeted postoperative aortic dimensions. The 3D patch design is transformed into a 2D geometry for intraoperative use. As our group pursues a preoperative patch-planning strategy for arch reconstruction, it is critical to know how the different aspects of the arch remodel and grow over time. There are many factors involved in the final arch calibre including patch remodelling, ongoing haemodynamic factors, potential underlying vascular wall abnormalities and residual ductal tissue. With the developing patch-planning workflow, it is possible to target *z*-score zero throughout the arch or to plan areas of the arch to have *z*-score larger than zero if that facilitates late outcome of a normal arch size. Therefore, this evaluation of the late arch geometry compared to the initial post-reconstruction size is essential to understand the precise target size for each aspect of the arch at initial reconstruction. Initial arch reconstruction between *z*-score 0 and +1 resulted in maintenance of that *z*-score size at late follow-up and this will be our initial size goal for our AAR patch-planning workflow. To facilitate this workflow, the importance of cross-sectional imaging in all age groups including neonates cannot be overstated. Preoperative CCT or CMR provides the substrate for detailed 3D segmentation and modelling and the necessary resolution for accurate patch planning. Precise preoperative patch sizing will more effectively achieve targeted pressurized aortic dimensions both perioperatively and long-term, which will ultimately lead to optimal patient outcomes with reduced reintervention rates and improved flow dynamics. Furthermore, postoperative cross-sectional imaging in the early and late periods will allow tracking of arch growth and haemodynamics over time but also provide valuable information for iteration of the preoperative workflow.

Our study found AAR with patch augmentation to be a safe operation with extremely high overall and transplant-free survival with reasonable postoperative LOS. Reintervention was performed in 33% of patients, with surgical correction most common in DKS patients and endovascular correction most common in non-DKS patients. Notably, a trend towards higher rate of reintervention was shown with decreasing AIsth initial postoperative repair *z*-score. Over 50% of reinterventions occurred within 6 months of index operation, likely partially attributable to arch undersizing at initial repair. An appreciable percentage of patients (26%) required intervention for developed LVOTO, which may be due to intrinsic pathophysiological processes in this congenital cohort. Future studies may elucidate prevention methods to minimize redo surgical and catheterization risk.

### Limitations

This study would have included almost 9 times more patients if TTE provided trustworthy arch measurements over 1 year of age. Unfortunately, we found that only cross-sectional imaging is adequate for longer follow-up arch dimension analysis. Prior authors have demonstrated poor correlation between TTE and cross-sectional imaging modalities in regard to aortic arch measurements, especially with increasing age, likely due to difficulty in achieving adequate acoustic windows with increasing body size [18–19]. The most thorough report of arch growth would be derived from patients with cross-sectional imaging at both timepoints (P1 and P3); however, this is a very limited population. Given improved aortic arch assessment in neonates and infants, the authors chose to include patients with TTE measurements at P1 as long as the patient was under 1 year of age at the time of imaging and excluded patients without CCT/CMR at P3. The cross-sectional imaging indications for these patients varied and were retrospectively utilized with limitations that our patient cohort is a fraction of all AAR patients during the study period and may not represent the general population of patients undergoing patch-augmented AAR, introducing an unadjusted selection bias. Patients with cross-sectional imaging may have a bias towards those with issues, highlighted by 44% of DKS patients having an arch reintervention. Given the small sample size (i.e. low statistical power) and lack of homogeneity among the cohort, other confounding patient variables such as HLHS and LVOTO may have potentially affected longitudinal aortic growth but were unable to be accounted for using statistical analyses. The portion of the cohort that received DKS was small and varied between neonates undergoing DKS and others receiving DKS at the Glenn stage. Given the small sample size across both DKS and non-DKS groups, our study was not powered to detect correlations between longitudinal aortic growth and functional/clinical outcomes.

## CONCLUSIONS

Over a mid-term follow-up, aortic longitudinal growth after patch-augmented AAR to *z*-score ±1 was proportional compared to the normal population. Initial repair size impacted mid-term growth, especially along the patched arch and AIsth, with potential for functional consequences. AIsth undersizing prevailed mid-term and trended towards a higher reintervention rate. Initial arch reconstruction between *z*-score 0 and +1 resulted in maintenance of that *z*-score size at late follow-up. Overall, it is crucial to achieve targeted aortic sizing at index operation to maintain appropriate aortic dimensions over time and reduce reintervention risk with specific focus on the AIsth.

## Supplementary Material

ivad210_Supplementary_Data

## Data Availability

All relevant data are within the manuscript and its Supporting Information files.

## ABBREVIATIONS

AA: Ascending aorta
AAR: Aortic arch reconstruction
AIsth: Aortic isthmus
AVV: Atrioventricular valve
BSA: Body surface area
CCT: Cardiac computed tomography
CI: Confidence interval
CMR: Cardiac magnetic resonance
DA: Descending aorta
DKS: Damus-Kaye-Stansel
DTA: Distal transverse arch
ECHO: Echocardiography
EDV: End-diastolic volume
HAA: Hypoplastic aortic arch
ICU: Intensive care unit
LOS: Length of stay
LVOTO: Left ventricular outflow tract obstruction
PTA: Proximal transverse arch
TTE: Transthoracic echocardiography
