# Supplementary material for: Aortic growth after arch reconstruction with patch augmentation: a 2-decade experience
Source: Interdiscip Cardiovasc Thorac Surg. 2023 Dec 22;37(6):ivad210. doi: 10.1093/icvts/ivad210 (PMC10752579; doi:10.1093/icvts/ivad210)
Supplement: ivad210_Supplementary_Data [file ivad210_supplementary_data.pdf]

Supplementary Materials for

Aortic Growth After Arch Reconstruction with Patch Augmentation: A Two Decade Experience

Dominic P. Recco; Shannen B. Kizilski; Reena M. Ghosh; Breanna Piekarski; Ashwin Prakash; David M. Hoganson*

*Corresponding author: David.Hoganson@cardio.chboston.org

Supplementary Tables

Table S1: Longitudinal Aortic Diameter Measurements

Patient Group	Aortic Segment	Diameter at 0-3 mo (cm)	Diameter at >12 mo (cm)	p-value	Z-score at 0-3 mo	Z-score >12 mo	p-value
DKS	Ascending Aorta (n=9)	1.20 [0.95, 1.30]	1.88 [1.32, 2.24]	0.004	0.36 [-0.06, 1.45]	0.05 [-0.57, 3.69]	0.570
	Proximal Transverse Arch (n=11)	0.95 [0.90, 1.10]	1.80 [1.67, 1.89]	<0.001	0.94 [0.35, 1.56]*	2.47 [-0.12, 3.60]*	0.206
	Distal Transverse Arch (n=16)	0.78 [0.68, 0.89]	1.39 [1.06, 1.68]	<0.001	-0.09 [-0.91, 0.58]	0.71 [-0.97, 2.34]	0.088
	Aortic Isthmus (n=14)	0.62 [0.55, 0.75]	1.02 [0.70, 1.30]	<0.001	-0.08 [-0.97, 0.43]	-0.29 [-1.51, 1.17]	0.761
	Descending Aorta (n=11)	0.55 [0.55, 0.60]	0.81 [0.73, 0.98]	0.003	N/A	N/A	N/A
Non-DKS	Ascending Aorta (n=25)	0.79 [0.69, 0.96]	1.67 [1.37, 2.23]	<0.001	-0.99 [-1.37, 1.08]	0.51 [-1.35, 1.44]	0.510
	Proximal Transverse Arch (n=24)	0.72 [0.65, 0.85]	1.70 [1.43, 1.94]	<0.001	-0.09 [-0.86, 1.03]	1.52 [0.29, 2.24]*	<0.001
	Distal Transverse Arch (n=34)	0.58 [0.46, 0.77]	1.38 [1.13, 1.66]	<0.001	-0.95 [-2.06, 1.07]*	-0.04 [-0.84, 0.53]	0.048
	Aortic Isthmus (n=32)	0.52 [0.42, 0.62]	1.13 [0.96, 1.28]	<0.001	-0.54 [-1.68, 0.70]	-0.33 [-1.22, 0.48]	0.278
	Descending Aorta (n=17)	0.54 [0.50, 0.65]	0.83 [0.70, 1.33]	<0.001	N/A	N/A	N/A

* denotes z-score is statistically different from z-score zero

Abbreviations: DKS: Damus-Kaye-Stansel.

Table S2: Longitudinal Aortic Z-Scores Stratified By Initial Postoperative Z-Score

Patient Group	Initial Z-score Grouping	Aortic Segment	Z-score at 0-3 mo	Z-score >12 mo	p-value
DKS	z-score <-1	AA (n=0)	N/A	N/A	N/A
		PTA (n=0)	N/A	N/A	N/A
		DTA (n=4)	-1.74 [-2.18,-1.36]	-0.78 [-1.12, 0.04]	0.125
		AIsth (n=3)	-1.39 [-2.28, -1.32]	1.17 [0.17, 1.52]	0.250
	-1≤ z-score ≤1	AA (n=5)	-0.05 [-0.14, 0.08]	0.05 [-0.61, 3.28]	0.625
		PTA (n=6)	0.39 [0.16, 0.49]	1.11 [-0.41, 2.47]	0.313
		DTA (n=9)	-0.09 [-0.48, 0.25]	1.04 [-0.16, 3.06]	0.250
		AIsth (n=9)	-0.05 [-0.28, 0.43]	-0.48 [-1.64, -0.30]	0.164
	z-score >1	AA (n=4)	1.47 [1.30, 1.96]	1.22 [-0.53, 4.76]	1
		PTA (n=5)	1.61 [1.40, 2.19]*	3.17 [1.75, 4.40]	0.438
		DTA (n=3)	1.46 [1.12, 2.60]	1.04 [-1.58, 2.86]	1
		AIsth (n=2)	2.40 [1.18, 3.63]	2.55 [-0.17, 5.27]	1
Non-DKS	z-score <-1	AA (n=11)	-1.38 [-1.65, -1.29]*	-1.19 [-2.46, 0.55]	0.365
		PTA (n=6)	-1.85 [-3.02, -1.31]*	-0.25 [-1.21, 0.43]	0.063
		DTA (n=16)	-2.25 [-2.96, -1.36]*	-0.36 [-1.10, 0.28]	<0.001
		AIsth (n=11)	-1.86 [-2.59, -1.67]*	-1.33 [-2.52, -0.47]*	0.019
	-1≤ z-score ≤1	AA (n=7)	0.07 [-0.90, 0.66]	0.94 [-0.67, 1.52]	0.688
		PTA (n=12)	-0.09 [-0.42, 0.65]	1.16 [0.70, 1.66]*	0.002
		DTA (n=9)	-0.40 [-0.87, 0.15]	0.01 [-0.34, 0.33]	0.496
		AIsth (n=16)	-0.35 [-0.60, 0.36]	-0.10 [-0.39, 0.56]	0.642
	z-score >1	AA (n=7)	1.67 [1.22, 1.73]*	1.41 [0.61, 2.56]	0.688
		PTA (n=6)	1.93 [1.32, 2.87]*	2.42 [2.19, 2.76]*	0.156
		DTA (n=9)	1.56 [1.17, 1.84]*	0.04 [-0.44, 1.57]	0.027
		AIsth (n=5)	2.03 [1.34, 2.27]*	0.49 [-0.81, 2.17]	0.438

* denotes z-score is statistically different from z-score zero

Abbreviations: AA: ascending aorta; AIsth: aortic isthmus; DKS: Damus-Kaye-Stansel; DTA: distal transverse arch; PTA: proximal transverse arch.

Supplementary Figures

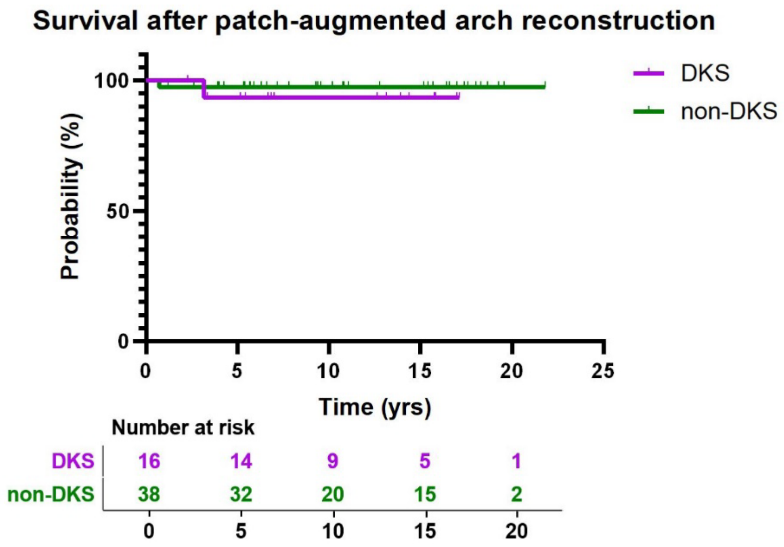

Figure S1: Kaplan-Meier survival curves for patients undergoing Damus-Kaye-Stansel (DKS) and non-DKS aortic arch reconstruction with patch augmentation.

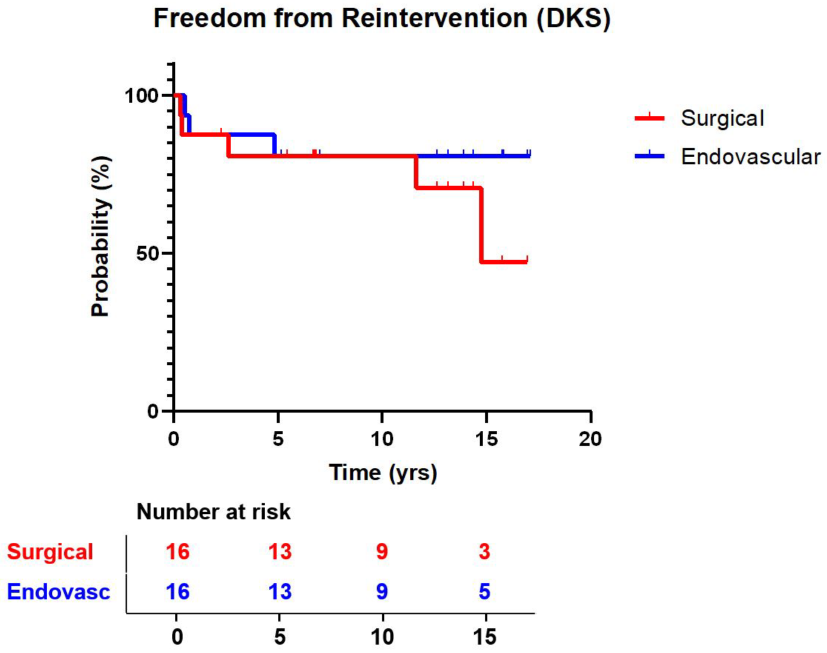

Figure S2: Kaplan-Meier freedom from surgical and endovascular reintervention for patients who underwent patch-augmented aortic arch reconstruction as part of the Damus-Kaye-Stansel (DKS) procedure.

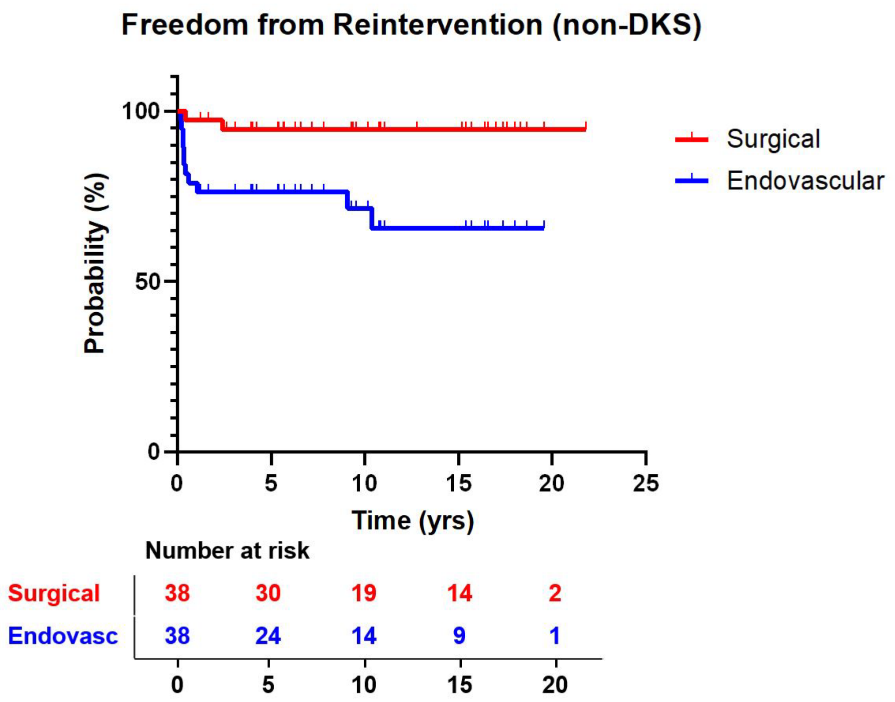

Figure S3: Kaplan-Meier freedom from surgical and endovascular reintervention for patients who underwent patch-augmented aortic arch reconstruction, excluding patients who underwent Damus-Kaye-Stansel (DKS) anastomosis.
